# Lack of variant specific CD8+ T-cell response against mutant and pre-existing variants leads to outgrowth of particular clones in acute hepatitis C

**DOI:** 10.1186/1743-422X-10-295

**Published:** 2013-09-28

**Authors:** Axel Ulsenheimer, Gláucia Paranhos-Baccalà, Florence Komurian-Pradel, Bijan Raziorrouh, Peter Kurktschiev, Helmut M Diepolder, Reinhart Zachoval, Michael Spannagl, Maria-Christina Jung, Norbert H Gruener

**Affiliations:** 1 Department of Internal Medicine II, Klinikum Großhadern, University of Munich, Marchioninistrasse 15, Munich, 81377, Germany; 2 Institute of Immunology, University of Munich, Munich, Germany; 3 Emerging Pathogens Laboratory, Fondation Mérieux, Lyon, France; 4 Laboratory of Immunogenetics and Molecular Diagnostics, Munich, Germany

**Keywords:** Acute hepatitis C, Quasispecies, T cell, Escape mutation, Epitope

## Abstract

**Background:**

CTL escape mutations have been described during acute hepatitis C in patients who developed chronic disease later on. Our aim was to investigate the mutual relationship between HCV specific CD8+ T cells and evolution of the viral sequence during early acute HCV infection.

**Results:**

We sequenced multiple clones of NS3 1406 epitope in 4 HLA-A*02 patients with acute hepatitis C genotype 1b infection. Pentamers specific for the variants were used to monitor the corresponding CD8+ T cell response. We observed outgrowth of mutations, which induced only a weak and thus potentially insufficient CD8+ T cell response. In one patient we observed outgrowth of variant epitopes with similarities to a different genotype rather than *de novo* mutations most probably due to a lack of responsiveness to these likely pre-existing variants. We could show that in acute hepatitis C CTL escape mutations occur much earlier than demonstrated in previous studies.

**Conclusions:**

The adaption of the virus to a new host is characterized by a high and rapid variability in epitopes under CD8+ T cell immune pressure. This adaption takes place during the very early phase of acute infection and strikingly some sequences were reduced below the limit of detection at some time points but were detected at high frequency again at later time points. Independent of the observed variability, HCV-specific CD8+ T cell responses decline and no adaption to different or new antigens during the course of infection could be detected.

## Background

Hepatitis C virus (HCV) causes acute and chronic hepatitis. The mechanisms that determine progression to chronic infection and outcome of HCV infection are not well understood. It is generally assumed that strong cellular immune responses are associated with viral clearance in acute hepatitis C infection [1-3]. Outcome of HCV infection is usually determined during the first weeks of acute infection. Recent data evidence selective pressure on HCV mediated through the HLA class I molecules and indicate that CD8+ T cell selection pressure influences viral evolution. However, there have been no studies in humans examining sequence evolution and contribution of CD8+ T cells during the early phase. The study by Tester et al. [4] followed two individuals acutely infected from a single source. An escape mutation was observed in the recipient who did not spontaneously resolve infection. Cox et al. [5] defined escape mutations in multiple CTL epitopes in eight acutely infected individuals. In a third study by Urbani et al. [6], escape mutations were found 1 and 3 months after onset of acute disease. Timm et al. [7,8] described escape mutations within epitope HLA B8 1395. The sequence in these studies usually was determined several months after acute disease. Several studies in the chimpanzee model were able to analyze very early viral evolution in acute HCV infection and found a correlation between outgrowth of escape mutations and the clinical outcome [9,10]. Although these studies constitute a critical mass of evidence for CTL escape mutations in HCV infection many questions, including timing, variability and whether these sequence changes represent outgrowth of particular clones of pre-existing quasi species or *de novo* mutations, remain to be answered.

In CTL epitope regions, especially of genotype 1b, gene diversity is significantly higher in NS3 than in other proteins [8,11]. We therefore investigated 4 patients during acute infection with genotype 1b and followed their response and evolution of variants within NS3 1406 epitope very early after onset of symptoms. Our results demonstrate that NS3 1406-specific, IFN-γ-secreting T cells can exert immune pressure resulting in extinction of certain strains. We observed outgrowth of mutations, which induced only a weak and thus potentially insufficient CD8+ response. In one patient we observed outgrowth of variant strains, which were more similar to sequences from a different genotype rather than random *de novo* mutations most likely due to a lack of responsiveness to these pre-existing strains. HCV-specific CD8+ T cell responses induced very early during infection seem to be unable to adapt to different or new antigens during the course of infection.

## Results

### Viral evolution within NS3 1406 epitope and corresponding T cell response in patients with acute hepatitis C

**Figure 1:**
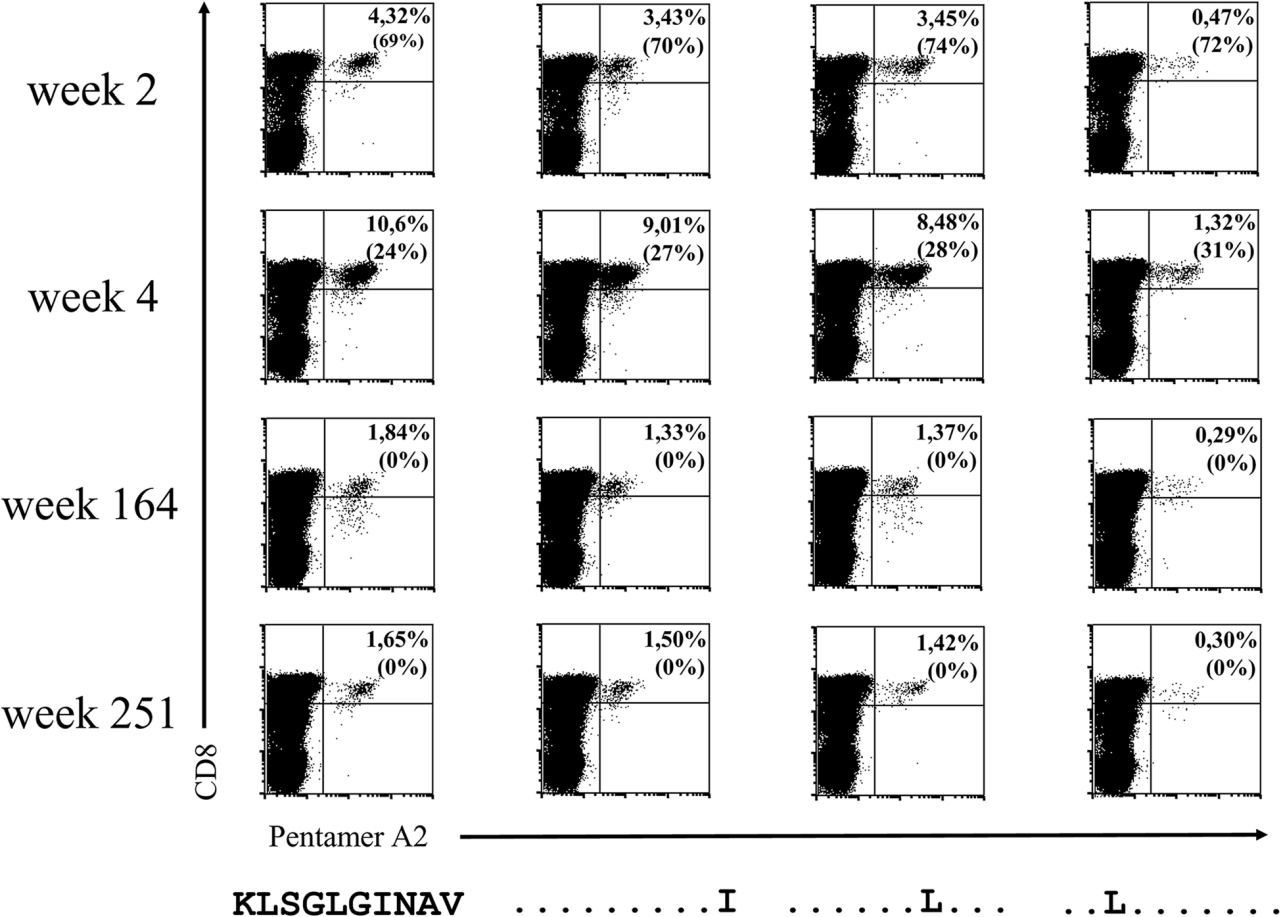
**Direct ex vivo peripheral blood frequency of HCV specific CD8+ T cells by ex vivo pentamer staining in patient 1.** Four different time points at week 2, 4, 164 and 251 after onset of acute disease are given. Pentamers with specifity for KLSGLGINAV, KLSGLGINAI, KLSGLGLNAV and KLLGLGINAV were used. The percentages of NS3 1406 specific CD8+ T cells/CD8+ T cell total are given. The percentages of CD38+ specific CD8+ T cells/NS3 1406 specific CD8+ T cells are given in brackets.

The NS3 region of the HCV genome including the NS3 1406 epitope was amplified by PCR and multiple clones were sequenced. We focused on the well-characterized CD8 T-cell epitope NS3 1406–15 with HLA-A0201* restriction, which has already been described in the context of escape mutations [4,11]. In patient 1, the sequence was KLSGLGINAV in 12 of 12 clones at the beginning of acute disease (week 1). At that time 4.32% of CD8+ T cells were specific for KLSGLGINAV. At the same time, 3,43% CD8+ T cells could be stained with pentamer KLSGLGINAI, 3.45% with pentamer KLSGLGLNAV and 0.47% with pentamer KLLGLGINAV. However, these sequences were not detected before week 2 (KLSGLGINAI), week 4 (KLSGLGLNAV) and week 7 (KLLGLGINAV) (Additional file 1: Table S1). At week 2, mutations developed at position 1415 in 16 of 16 clones (KLSGLGINAI). At week 3, the original sequence KLSGLGINAV again occurred in all 13 clones, at this time with a mutation in the flanking region. One week later, the sequence of week 2 was detected again in 7 of 11 clones, while in 4 of 11 clones a novel sequence (KLSGLGLNAV) was found. Pentamer staining at week 4 revealed the highest percentage of NS3 1406-specific CD8+ T cells in comparison to all other time points. Over time, staining at the different time points revealed the highest direct ex vivo frequencies for KLSGLGINAV followed by KLSGLGINAI, KLSGLGLNAV and KLLGLGINAV. This relative proportion of these variant-specific CD8+ T cells remained unchanged during the course of disease despite a high fluctuation of sequences and viral load found at the different time points (Figure 1). The specific cells were also stained with anti-CD38. Expression of CD38 dropped in all variants over time until week 251. In week 5, the sequence of week 2 KLSGLGINAI was again the dominant sequence in all 14 clones tested. At week 7, a previously undetected sequence (KLLGLGINAV) was found in 10 of 12 clones. At week 34, KLSGLGLNAV was the only sequence found in 12 of 12 clones. Between week 6 and week 34, no amplification was possible due to a low viral load. Given the high variability within the NS3 1406 epitope, there were fluctuating HCV RNA levels during acute infection. At week 1, a high viral load was found, rapidly dropping within 7 days and again increasing ten-fold during another 7 days (Additional file 1: Table S1). Another 3 weeks later, the viral load again dropped 100-fold. Within the following week, a new 100-fold increase was observed. Then a 1000-fold decrease occurred in the next 2 weeks and viral load remained low for several months. By week 37, another increase of the viral load to 4046157 cp/ml was observed. At that time, therapy with pegylated interferon and ribavirin for 48 weeks was started; due to a sustained virological response no virus was detectable in the follow-up period.

However, 164 and 251 weeks after acute disease, still significant numbers of NS 3 1406-specific CD8+ T cells were detectable, again with the same relative proportion.

In patient 2, HCV RNA was positive at three time points. At week 2, KLSGLGINAI was dominant in 13 of 13 clones. One week later, KLVALGINAV was the main sequence in 22 of 26 clones. This sequence resembles the genotype 1a sequence. After this, no virus was detectable until week 13. At that time, the initial sequence of week 1 was again the dominant sequence in 17 of 17 clones (Additional file 2: Table S2a).

In patient 2, the viral load in week 2 was 233477 cp/ml, remaining identical in week 3. In week 4, no virus was found. In week 13, again 71047 cp/ml were detectable.

**Figure 2:**
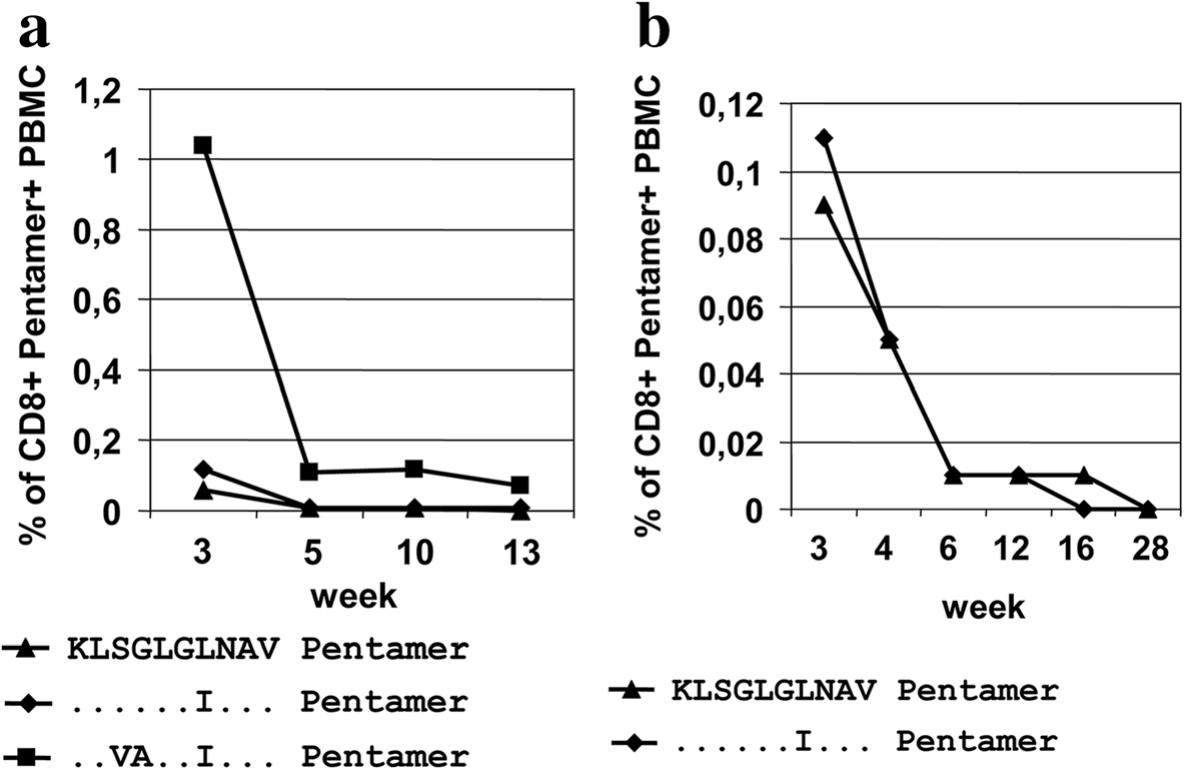
**Peripheral blood frequency of HCV specific CD8+ T cells by ex vivo pentamer staining.** Frequencies in patient 2. Frequencies in patient 3. Different time points after onset of acute disease are given. Pentamers with specifity for KLSGLGINAV, KLSGLGLNAV, KLVALGINAV were used.

At week 3, in patient 2, 1% of the CD8+ T cells specific for NS3 1406 KLVALGINAV and low frequency of KLSGLGINAV and KLSGLGLNAV could be stained. During further follow-up, frequencies dropped to 0.1% for NS3 1406 KLVALGINAV and below detection limit for KLSGLGINAV and KLSGLGLNAV (Figure 2). Patient did not receive treatment and was lost to follow-up.

In patient 3, a mixed population was found with KLSGLGLNAV in 21/26 clones and KLSGLGINAI in 4/26 clones at week 3 a. One week later, KLSGLGLNAV was found in 12/23 clones and KLSGLGINAI in 11/23 clones. At that time, therapy was started and no virus was detectable during follow-up (Additional file 2: Table S2b).

In patient 3, 833400 cp/ml were found in week 2. The viral load dropped to 17335 cp/ml in week 3. At that time, therapy was started and no virus was detectable in the follow-up period.

In patient 3, only low frequencies of KLSGLGINAV and KLSGLGLNAV specific CD8+ T cells were detectable at week 3. No NS 3 1406-specific CD8+ T cells were detectable thereafter (Figure 2).

In patient 4, sequencing of NS3 1406 was done at week 2, week 15 and week 22. No mutations were observed during this period. At all 3 time points, KLSGLGINAI was found as main sequence. At the other time points, no virus was detectable. HCV RNA was detectable in week 4 but no amplification of the epitope was possible at that time (data not shown).

In patient 4, the viral load was low in week 1. No virus was detectable in week 9. In week 2, 15 and 22, a low viral load was detectable. After week 22, no virus was found.

In patient 4, no NS 3 1406-specific CD8+ T cells were detectable at any time. An immediate therapy with pegylated interferon was started in week 2 and a sustained virological response was achieved in this patient.

### Viral evolution within NS3 1317–1423 outside NS3 1406

Since variability of epitopes can be influenced by their flanking regions, we also analyzed the variability of the sequences outside NS3 1406. Sequence data of NS3 1317–1423 revealed that the high variability detected within the epitope 1406 in patient 1 could not be detected within NS3 1317–1423. In contrast the sequence was very stable over time (Additional file 2: Table S2c). Only at the time when the viral load came up again (week 37) a mutation within patient’s HLA-A02* restricted NS3 1321–35 epitope (TDSTSILGIGTVLDQ) to NS3 1321–35 epitope (IDSTSILGIGTVLDQ) was observed. Another mutation was found at position 1282 V to I, which represents the flanking region of patient’s HLA-A02* restricted NS3 1284–1296 epitope [12].

Also in patient 2 and 3 sequence variations were found within known CTL epitopes, NS3 HLA-A02* 1321–35 [13], HLA-B35* NS 3 1359–67 [14], NS3 1382-1397 [5], HLA-B08* NS3 1395–1403 [15] or the direct flanking region of known CTL epitope HLA-C14*1371-80 [5]). In patient 2 at week 2 patient’s HLA-A02* restricted NS3 1321–35 TDSTSILGIGTVLDQ was found, at week 3 a sequence variation at position 1323 was detected, NS3 1321–35 TDATSILGIGTVLDQ. 6 weeks later again the initial sequence came up. Mutations were also found in epitopes which did not match with patient’s HLA. NS 3 1359–67 epitope HSNIEEVAL was sequenced at week 2. Amino acid exchange at position 1360 could be detected at week 3 HPNIEEVAL. Again at week 9 the initial sequence was found. The same phenomenon was observed within NS3 1382–97. IETIKGGRHLIFCH (week 2) to LEVIKGGRHLIFCH (week 3) and back to IETIKGGRHLIFCH (week 9). At position 1369, the flanking region of NS3 1371–80, amino acid exchange N (week 2) to T (week 3) to N (week 9) was found. In patient 3 matching HLA-A02* restricted NS3 1321–35 TDSTTILGIGTVLDQ and NS3 1321–35 TDSTSILGIGTVLDQ were found at week 3 and 4. Another sequence variation was observed within patient’s HLA-B08* restricted NS3 1395–1403 epitope. HSKRKCDEL and HSRKKCDEL were present at week 3 and 4. This mutation has been described frequently in the presence of HLA B*08 allele [7,16]. At position 1370, the flanking region of NS3 1371–80, amino acid exchange I (week 3) to T (week 4) was found.

Thus all sequence variations in NS3 were observed within known CTL epitopes or in their flanking regions. The ongoing variation was seen mainly in patient’s HLA restricted epitopes. Only in patient 2 variations were found in patient’s HLA non-restricted epitopes (NS3 1359–67, NS3 1382–97). For the HLA-C14 restricted NS3 1371–80 epitope the patient’s corresponding HLA was unknown. At least in patient 2 the sequence variation found might reflect different pre-existing strains since the main sequence at week 2 and 9 were identical. In addition the main sequence found in week 3 differed at 8 positions from the main sequence at week 2/9. Furthermore the main sequence with the KLVALGINAV epitope is usually found in genotype 1a infected patients in contrast to the KLSGLGINAI sequence usually found in genotype 1b infected patients [17]. In addition variations were found in patient’s HLA non-restricted epitopes indicating different strains rather than ongoing selection pressure. As another variation KLSGLGLNAV occurred in 4/22 clones either as a consequence of mutations from week 2 sequences or also as already pre-existing so far undetected variation. The complete reduction of the main sequence below the detection threshold within a few days and reappearance at a later time point might be caused by the ongoing selection process in acute infection and the fluctuations in viral load, which could drop below the detection limit of our methods. This was observed in patient 1 as well as in patient 2.

### Induction of γ-interferon production of PBMC after stimulation with different NS3 1406 epitopes

**Figure 3:**
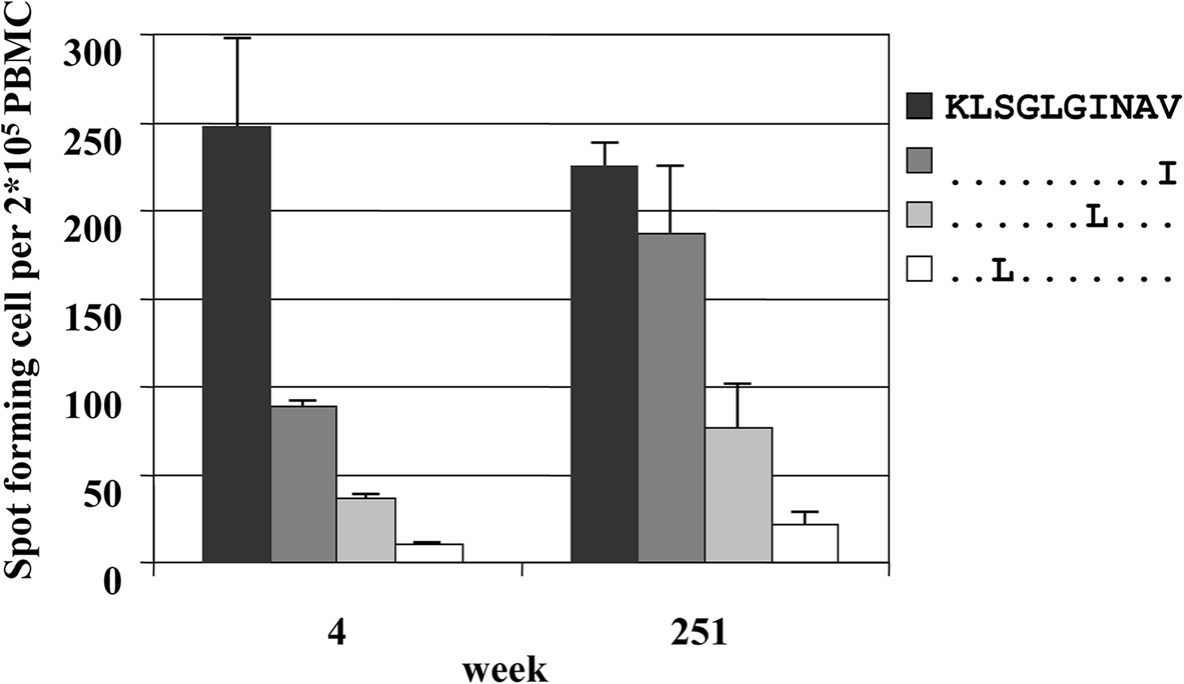
**Enzyme-linked immunospot (ELISPOT) results for patient 1.** Peripheral blood mononuclear cells (PBMC) from patient 1 were stimulated with 20 μg/ml of NS3 1406 peptides as indicated for 48 h, and the interferon-gamma production was measured in the ELISPOT assay as described in material and methods. Each bar represents the specific spots/2 × 10^5^ PBMC. Two different time points (4 and 251 weeks after acute infection) are shown. The error bars represent the standard error of the mean.

To further address the question whether the mutations seen in acute hepatitis C infection resemble escape mutations we investigated the ability to induce production of γ-IFN of PBMC early (week 4) and late (week 251) after acute infection in patient 1. We were not able to test other time points due to lack of patient material. The production of γ-IFN of PBMC after stimulation with wild type or mutant peptides was tested with ELISpot assay as described in material and methods. After stimulation we found that the initial sequence KLSGLGINAV was able to induce high amounts of γ-IFN (> 200 specific spots/2 × 10^5^ PBMC). KLSGLGLNAV, which was found at week 37 in all clones induced significantly less γ-IFN (Figure 3).

### T cell clones and cross reactivity

**Figure 4:**
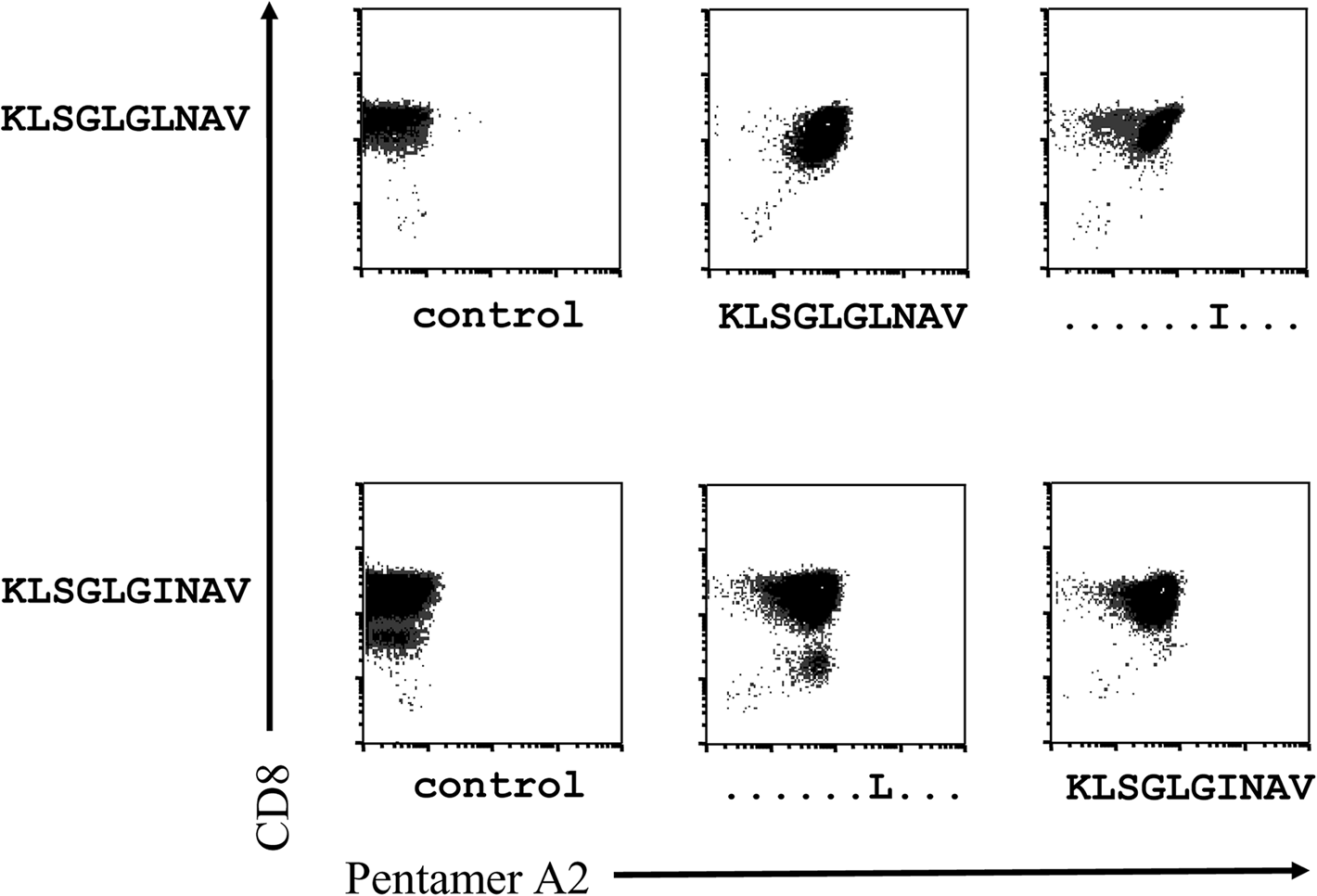
**64 weeks after disease onset of patient 1 and after successful interferon therapy clones were generated with specificity for KLSGLGLNAV and KLSGLGINAV as it is indicated on the y-axis.** The different clones were tested with pentamers as indicated on the x-axis.

CD8+ T cell clones specific for KLSGLGINAV and KLSGLGLNAV were generated. Staining of these T cell clones revealed cross reactivity of pentamer KLSGLGINAV and KLSGLGLNAV. Staining of the clone KLSGLGINAV with pentamer KLSGLGLNAV and vice versa was possible (Figure 4).

## Discussion

In our study of sequence variations within NS3 1406 epitope during early acute HCV, we observed several features, which potentially contribute to the development of chronic infection. We demonstrated an extremely high variability within this epitope during the first weeks. A particular characteristic were significant changes in the frequency of different variants with some being reduced below the limit of detection at some time point but being detected at high frequency again a few days later on. No new T cell responses were detected in response to mutant peptides. The mutated variants found were characterized by a reduced capacity to induce IFN-γ. The rapid and high mutation rate was observed mainly within epitope NS3 1406. In contrast, NS3 1317–1423 was characterized by rare mutations, most of them detectable within patient’s HLA restricted known epitopes. Not only the original sequence but also variant sequences found during early infection dropped below the limit of detection transiently and reappeared during further follow-up. This alternation of sequences was observed over several weeks in patient 1. In patient 1, at week 3 the sequence KLSGLGINAV, which has been detected at week 1 as main sequence in 12/12 clones, occurred again in 13/13 clones; however, this time with a mutation in the flanking region. Interestingly, at week 2, this sequence could not be detected in 16 clones analyzed. Therefore, this mutation in the flanking region of the epitope may favor the transient reoccurrence of the original sequence i.e. by changing the pattern of proteasome digestion. This mechanism has recently been described by Kimura et al. in chimpanzees [18]. A similar phenomenon of vanishing and reoccurrence of sequences was seen in patient 2. However, the sequences found at different time points resembled index sequences of different genotypes and might reflect rather pre-existing variations than random de-novo mutations. In contrast to patient 1, the sequence that reoccurred in patient 2 was not recognized by specific CD8+ T cells at all. NS3 1406-specific CD8+ T cells were found against the presumably genotype 1a sequence but not against the putative genotype 1b sequence. This is remarkable since this suggests that CTL escape is not the only selection criteria. Despite mutations or pre-existing variants that result in the production of new or different potential antigens, our data give evidence that no new CD8+ T cell responses were generated against the new variants that were found during infection. This interpretation is supported by different observations. Pentamer staining was positive for all variants in patient 1 at the earliest available time point in week 2. It remains unclear if these different specific CD8+ T cells have been induced very early or if this reflects cross reactivity between the variants. The proportion of these variant-specific CD8+ T cells remained stable over a long period of time, which rather indicates cross reactivity and a lack of new variant-specific CD8+ T cells over time. In addition, the activation marker CD38 dropped during the course of infection in all pentamer-positive CD8+ T cells in patient 1 despite a high variability in sequence and viral load during this phase. It has been reported earlier by us that HCV-specific CD8+ T cells are induced but not sustained [19]. In extension to our former observation, this holds true despite the proven high variability of sequence and viral load. Furthermore, using specific CD8+ T cell clones from patient 1, we demonstrated cross reactivity of the pentamer staining for variant epitopes. In patient 2, we found two different strains during the course of acute disease. These sequences are usually found in different genotypes (1a and 1b). One possible explanation is that these variants correspond to pre-existing variants rather than random de-novo mutations. However, specific CD8+ T cells were only observed against the genotype 1a epitope but not the genotype 1b epitope. All these findings support a lack of generation of new variant-specific CD8+ T cells against mutant or pre-existing variants during acute HCV infection despite a high variability in sequence and viral load. This phenomenon has been observed by others as well [5,6]. Explanation for this observation had been that original antigenic sin [20] may be responsible for the lack of response to mutant sequences or that the mutant sequences cannot be processed effectively for presentation. Differences were observed in terms of the functional capacity of the different sequences. The initial sequence in patient 1 was capable of inducing the highest amounts of IFN-γ. Functionality with regard to IFN-γ production remained high over time as we observed similar IFN-γ levels after 251 weeks in patient 1. The mutated variants were less potent or did not induce IFN- γ- production at all. The variants that remained and were selected by the virus were either less efficient in inducing IFN-γ production (patient 1) or were not recognized by CD8+ T cells due to a lack of priming of a response specific for the corresponding variant (patient 2).

Several limitations need to be considered in this study. The methods used might not be sensitive enough to detect certain minor variants of the *quasispecies*. Thus, it is difficult to distinguish between levels below the detection limit or complete disappearance of a variant. Additionally, the sequence of the inoculum was unknown but could have provided valuable information on the pre-existing variants. Moreover our study mainly focused on a single epitope and its surrounding sequences. Therefore it remains to be determined if mutations and escape occurs as quickly at other CD8+ T cell epitopes.

Despite these limitations we had the opportunity, in contrast to former studies, to gain our data very early after acute disease and were thus able to find a high variability within a known CD8+ T cell epitope. This could also explain some of the different observations between our results and former studies. Kuntzen et al. [14] described two patients who progressed to chronicity without substantial escape in targeted epitopes. However, the earliest sequence analysis was done 2 and 2.5 months after acute infection. Thus, transient mutations with reappearance of former sequences could have been missed in this study. In addition, this study found that mutations outside envelope overall declined over the course of infection. Taking this observation and our data together, sequence analysis within the first weeks or even days after infection or acute disease may additionally help to elucidate the role of CD8+ T cell escape mutations in driving the evolution of HCV.

Outside the NS3 1406 epitope within NS3 1318–1423, which was the region sequenced in our study, a low variability was found. Mutations almost exclusively affected formerly described CTL epitopes or their flanking regions. The ongoing selection was observed in patient’s HLA restricted epitopes but not in patient’s HLA non-restricted epitopes. The only exception found of a variation in HLA non-restricted epitopes (NS3 1359–67, NS3 1382–97) in patient 2 can be either explained by pre-existing different strains or, in the absence of ongoing selecting immune pressure, reversion of a mutation to a more favourable sequence for the virus with respect to viral replication. Our findings indicate that the virus is very stable without immune pressure. Future longitudinal analysis of the whole genome during the early phase of acute infection should elucidate the hot spots like NS3 1406 epitope. A better understanding of these hot spots could be of outstanding importance for a vaccine development.

## Conclusions

In summary, we demonstrated that CTL escape mutations occur much earlier than previously demonstrated in acute HCV infection. The adaption of the virus to a new host is characterized by a high and rapid variability in epitopes under CD8+ T cell immune pressure. This adaption takes place during the very early phase of acute infection. Variants can drop below the limit of detection during the course of infection and reappear at later time points. Most strikingly, HCV-specific CD8+ T cell responses induced very early during infection seem to be unable to adapt to different or new antigens during the course of infection. Independent of the variability, the CD8+ T cell response is not sustained sufficiently. This phenomenon indicates that different complementary mechanisms are active in acute hepatitis C. Epitopes under immune pressure evade by mutations but can reoccur while the CD8+ response is already vanishing. A better understanding of this peculiar silencing of the CD8+ T cell response, which seems to be one of the hallmarks of acute hepatitis C, could be critical in further elucidating the pathogenesis of the chronic phase of the disease.

## Methods

### Patients

In this study the following patients were included: 4 patients with acute hepatitis C infection. Time point zero was defined as onset of symptoms and diagnosis of acute HCV infection. Acute hepatitis C was diagnosed by documented seroconversion to anti-HCV antibodies or all of the following: acute onset of hepatitis in previously healthy individual, aminotransferases at least 10× the upper limit of normal, exclusion of other infectious, metabolic, or toxic causes of hepatitis, recent exposure or source of infection identified. At different time points during the course of disease patient 1, 3 and 4 received antiviral treatment with subsequent viral elimination. Patient 2 did not receive antiviral treatment. For further clinical characteristics of the patients see Table 1. Further inclusion criteria were HLA-A0201 genotype and positive tetramer staining with the NS3 1406–1415 index pentamer. All patients gave informed consent to participate in the study and the protocol and the procedures of the study were conducted in conformity with the ethical guidelines of the Declaration of Helsinki. The study was approved by local ethical committee, University of Munich. None of the patients were enrolled in any previous studies.

**Table 1:** Patients characteristics

**ID**	**Age**	**Gender**	**Treatment duration**	**Antiviral treatment**	**Possible transmission**	**GPT IU/ml**	**Genotype**	**HLA A,B**
1	57	m	Week 38-93	Peg-interferon/ribavirin	Unknown	1181	1b	A2,A3,B7,Bw6
2	38	m	None	None	Sexual	1996	1b	A2,A26,B15,B51
3	63	m	Week5-31	Peg-interferon	Unknown	616	1b	A1,A2,B7,B8
4	46	f	Week2-28	peg-interferon	Medical	647	1b	A1,A2,B4,B51

### Preparation of peripheral blood mononuclear cells

Peripheral blood mononuclear cells (PBMC) were isolated by Ficoll-Hypaque density centrifugation (Biochrom, Berlin, Germany) of fresh heparinized peripheral blood. Briefly, PBMC were washed four times in phosphate buffered saline (PBS), counted and resuspended in 1,5 ml FCS supplemented with 10% DMSO. The maximum cell number per vial was 25 × 10^6^. Cells were kept at −80°C for 24 hours before being transferred to −196°C. Thawing was performed by inserting frozen probes in a prewarmed water bath at 37°C. After thawing cells were immediately washed in cell culture medium to remove residual DMSO and resuspended in tissue cultured medium (RPMI 1640 medium; Gibco, Grand Island, N.Y.) containing 2 mM L-glutamine, 1 mM sodium pyruvate, 100 U of penicillin per ml, 100 μg of streptomycin per ml and 5% human AB serum.

### HCV peptides and MHC class I pentamer staining on PBMC

Peptides (10mers) were synthesized by Proimmune (Oxford, UK) or EMC (Microcollections, Tübingen, Germany). Lyophilized peptides were reconstituted at 20 mg/mL in dimethyl sulfoxide (Roth, Karlsruhe, Germany) and were diluted to 1 mg/mL in RPMI 1640 medium (Biochrom, Berlin, Germany).

Class I pentamers specific for different variants of HLA-A02* restricted NS-3 1406 epitopes, which were detected in our patients during acute HCV (KLSGLGLNAV, KLLGLGINAV, KLSGLGINAI, KLSGLGINAV, KLVALGINAV) were synthesized by Proimmune (Oxford, UK).

PBMC were stained in 100 μl medium (RPMI, 5% AB serum, 2 mM glutamine, 50 U/ml Penicillin-Streptomycin) with 10 μl of PE- or APC conjugated MHC class I pentamer for 30 minutes at room temperature according to the manufacturer’s instructions. APC-conjugated anti-CD8, PerCP-conjugated anti-CD14, PerCP-conjugated anti-CD19, Viaprobe (Becton Dickinson), and FITC-conjugated anti-CD38 monoclonal antibodies (Becton Dickinson) were added for the last 20 minutes of incubation.

### Elispot assay

PBMC (2*10^5^/well) from patients were tested with respect to their interferon gamma production by a commercial Elispot Kit system (ELISpotPRO for human interferon-γ, MABTECH AB Büro Deutschland, Hamburg, Germany). The assay was performed on bulk PBMC according to the manufacturer’s instructions as previously described [3]. The spots were counted by an automated Elispot reader (EliSpot Reader System, Autoimmun Diagnostika GmbH, Straßberg, Germany). The following peptides were used for stimulation: 1. KLSGLGINAV, 2. KLSGLGINAI, 3. KLSGLGLNAV and 4. KLLGLGINAV.

### Generation of T-cell clone and specificity testing

PBMCs (5*10^4^/well) of patient 1 were incubated in 96-well U-bottom plates (TPP, Trasadingen, Switzerland) in the presence of HCV peptides (10 μg/ml) in 150 μl of tissue culture medium. On day six, recombinant interleukin 2 (IL-2) was added to a final concentration of 200 U/ml (IL-2 was a gift of Basel Institute for Immunology). On day 10 cells were cloned at 0,5 cells/well in the presence of 3*10^4^ irradiated PBMC per well, 200 U of IL-2 per ml, and 2 μg per ml of phytohemagglutinin (Murex Biotech Ltd., Kent, UK)). After 3 to 5 weeks clones were tested for specificity by pentamer staining as described above with minor modifications.

For expansion, T-cell clones were stimulated every 2 to 5 weeks with irradiated allogeneic PBMC in culture medium with 200 U/ml IL-2 and 2 μg/ml PHA. Culture medium was changed every 2–5 days.

### HLA typing

DNA was obtained from whole blood using the QIAamp DNA Blood Mini Kit following the manufacturer’s guidelines. HLA Class I typing was performed by PCR and/or direct DNA sequencing by the Laboratory of Immunogenetics and Molecular Diagnostics, University of Munich.

### HCV-RNA quantitation and genotyping

Sera were tested for HCV-RNA content and genotype by real-time polymerase chain reaction (PCR) of the 5′ HCV non-coding region as previously described [21,22]. Briefly, RNA (4 ml) extracted from 50 μl of serum by the QIAamp viral RNA kit (Qiagen S.A, Courtaboeuf, France) was reverse-transcribed with the ThermoScript™ Reverse Transcriptase kit (Gibco/BR, Cergy Pontoise, France) using RC21 primer. Real-time PCR were carried out with 2 ml of cDNA and RC1 and RC21 primers using the LC FastStart DNA Master SYBER Green I kit and the LightCycler™ apparatus (Roche Diagnostics, Meylan, France).

### Sequencing of autologous virus

Viral RNA was extracted from plasma samples using Qiagen (Hilden, Germany) vRNA extraction kit. The NS3 region was targeted for amplification by nested RT-PCR by using Titan one tube RT-PCR kit (Roche) and the Expand High Fidelity PCR system (Roche). Specific primers were designed for Genotype 1a and 1b based on alignments of available sequences from the public HCV Database (http://hcv.lanl.gov) [23]. PCR products were obtained with the following primers: primer 1- TTACGTATTCCACCTATGGC, primer 2 - GACACATCAAGACCCCGGTA, for the round of the RT-PCR following by primers: 3-GACACATCAAGACCCCGGTA, and 4-ATGAGTGCCACTCAACTGAC, purified and cloned in pCR-TOPO (Invitrogen). After cloning, amplified fragments were sequenced in both directions by using the BigDye Terminator version 1.1 cycle sequencing kit (Applied Biosystems) and ABI Prism 3100 genetic analyzer (PE/Applied Biosystem). Sequence analysis was carried out with Vector NTI suite (Invitrogen). The sequences were analyzed by using nucleotide blast (blastn) (http://blast.ncbi.nlm.nih.gov/Blast.cgi) and ClustalX (v1.4) for alignment with previously described sequences.

## Competing interests

The authors declare that they have no competing interests.

## Authors’ contributions

AU and GPB contributed equally to this work. AU, BR, PK, WS, HD and NG designed the experiments and analyzed the data. MS did the HLA Typing. GPB and FKP performed HCV RNA quantitation and sequencing. RZ and MCJ provided patient samples, clinical information and critically reviewed the manuscript. AU and NG wrote the manuscript. All authors read and approved the final manuscript.

## Supplementary Material

Additional file 1: Table S1 Evolution of NS3 1406 sequence and viral load in patient 1 at week 1, 2, 3, 4, 5, 7 and 37 of acute hepatitis C infection. Click here for file

Additional file 2: Table S2 **a**: Evolution of NS3 1317–1423 sequence in patient 1 at week 1, 2, 3, 4, 5, 7 and 37 of acute hepatitis C infection. **b**: Evolution of NS3 1317–1423 sequence in patient 2 at week 2, 3 and 9 of acute hepatitis C infection. **c**: Evolution of NS3 1317–1423 sequence in patient 3 at week 3 and 4 of acute hepatitis C infection. Click here for file
