# Supplementary material for: Lack of variant specific CD8+ T-cell response against mutant and pre-existing variants leads to outgrowth of particular clones in acute hepatitis C
Source: Virol J. 2013 Sep 28;10:295. doi: 10.1186/1743-422X-10-295 (PMC3849755; doi:10.1186/1743-422X-10-295)
Supplement: Additional file 1: Table S1 — Evolution of NS3 1406 sequence and viral load in patient 1 at week 1, 2, 3, 4, 5, 7 and 37 of acute hepatitis C infection. [file 1743-422X-10-295-S1.docx]

Additional file 1: Table S1: Evolution of NS3 1406 sequence and viral load in patient 1 at week 1, 2, 3, 4, 5, 7 and 37 of acute hepatitis C infection

viral load

**(10**^6^cp/ml)

clones tested

sequence evolution

timepoint

**(week)**

14/14

4/11

7/11

13/13

1/16

15/16

12/12

..**.**.......

......**L**...

..**L**.......

**.........I**

......**L**...

**.........I**

......**.**...

**....P....I**

**.........I**

**KLSGLGINAV**

37

7

5

4

3

2

1

1

4

32

40

18

3

33

12/12

2/12

10/12
