# Supplementary material for: Lack of variant specific CD8+ T-cell response against mutant and pre-existing variants leads to outgrowth of particular clones in acute hepatitis C
Source: Virol J. 2013 Sep 28;10:295. doi: 10.1186/1743-422X-10-295 (PMC3849755; doi:10.1186/1743-422X-10-295)
Supplement: Additional file 2: Table S2 — a: Evolution of NS3 1317–1423 sequence in patient 1 at week 1, 2, 3, 4, 5, 7 and 37 of acute hepatitis C infection. b: Evolution of NS3 1317–1423 sequence in patient 2 at week 2, 3 and 9 of acute hepatitis C infection. c: Evolution of NS3 1317–1423 sequence in patient 3 at week 3 and 4 of acute hepatitis C infection. [file 1743-422X-10-295-S2.doc]

ECHSTDSTSILGIGTVLDQAETAGARLVVLATATPPGSVTVPHSNIEEVALSNT

..R...................................................

......................................................

..................G...................................

......................................................

................................T.....................

......................................................

......................................................

...............A......................................

......................................................

......................................................

.........................................S............

.....................................................A

......................................................

......................................................

......................................................

....I.................................................

....I.................................................

....I.................................................

....I..............V..................................

....I.................................................

....IG................................................

.K..I.................................................

Week 1 (11/12)

Week 1 (1/12)

Week 2 (13/16)

Week 2 (1/16)

Week 2 (1/16)

Week 2 (1/16)

Week 3 (11/13)

Week 3 (1/13)

Week 3 (1/13)

Week 4 (4/11)

Week 4 (4/11)

Week 4 (1/11)

Week 4 (1/11)

Week 5 (14/15)

Week 5 (1/15)

Week 7

Week 37 (6/12)

Week 37 (1/12)

Week 37 (1/12)

Week 37 (1/12)

Week 37 (1/12)

Week 37 (1/12)

Week 37 (1/12)

Additional file 2: Table S2a: Evolution of NS3 1317-1423 sequence in patient 1

1320

1325

1330

1335

1340

1345

1350

1355

1360

1365

1370

NS3

GEIPFYGKAIPIETIKGGRHLIFCHSRKKCDELAA*KLSGLGINAV*AYYRGLDV

.....................................................

...................................*.........I*........

............................................I........

.......................................P....I........

............................................I........

..................................T*..........*........

...............R..................T..................

..................................T..................

...................................*.........I*........

.........................................L...........

............................................I........

............................................I........

...................................*.........I*........

..................G.........................I........

...................................*..L.......*........

............................................T........

.............................R.......L...............

...................................*......L...*........

.........................................L....H......

...............T.........................L...........

.........................................L...........

...........V.............................L...........

.........................................L...........

.........................................L...........

Week 1 (11/12)

Week 1 (1/11)

Week 2 (13/16)

Week 2 (1/16)

Week 2 (1/16)

Week 2 (1/16)

Week 3 (11/13)

Week 3 (1/13)

Week 3 (1/13)

Week 4 (4/11)

Week 4 (4/11)

Week 4 (1/11)

Week 4 (1/11)

Week 5 (14/15)

Week 5 (1/15)

Week 7 (9/12)

Week 7 (2/12)

Week 7 (1/12)

Week 37 (5/12)

Week 37 (1/12)

Week 37 (1/12)

Week 37 (1/12)

Week 37 (1/12)

Week 37 (1/12)

Week 37 (1/12)

Additional file 2: Table S2a

1375

1380

1385

1390

1395

1400

1405

1410

1415

1420

Week 2 (13/13)

Week 3 (18/26)

Week 3 (1/26)

Week 3 (1/26)

Week 3 (1/26)

Week 3 (1/26)

Week 3 (1/26)

Week 3 (1/26)

Week 3 (1/26)

Week 3 (1/26)

Week 9 (16/17)

Week 9 (1/17)

Additional file 2: Table S2b: Evolution of NS3 1317-1423 sequence in patient 2

1320

1325

1330

1335

1340

1345

1350

1355

1360

1365

1370

NS3

ECHSTDSTSILGIGTVLDQAETAGARLVVLATATPPGSVTVPHSNIEEVALSNT

......A....................................P........T.

......................................................

......A.........P..........................P........T.

......................................................

......A....................................P........T.

......A.............G......................P........T.

......................................................

......A.............G......................P........T.

......................................................

......................................................

...........D..........................................

GEIPFYGKAIPIETIKGGRHLIFCHSRKKCDELAA*KLSGLGINAI*AYYRGLDV

...........L.V.....................*..VA.....V*........

...................................*......L..V*........

...........L.V....................*...VA..F..*V........

..................................*.......L..*V........

.K.........L.V....................*...VA.....*V........

...........L.V....................*...VA.....*V........

..................................*.......L..*V........

...........L.V..............E.....*...VA..F..*V........

..................................*.......L..*V........

..................................*..........*.........

..................................*..........*.........

Week 2 (13/13)

Week 3 (18/26)

Week 3 (1/26)

Week 3 (1/26)

Week 3 (1/26)

Week 3 (1/26)

Week 3 (1/26)

Week 3 (1/26)

Week 3 (1/26)

Week 3 (1/26)

Week 9 (16/17)

Week 9 (1/17)

Additional file 2: Table S2b:

1375

1380

1385

1390

1395

1400

1405

1410

1415

1420

ECHSTDSTTILGIGTVLDQAETAGARLVVLATATPPGSVTVPHPNIEEVALSNI

......................................................

......................................................

......................................................

......................................................

.........S.................................S.........T

.........S............................A....S.........T

.........S.................................S.........T

.........S.................................S.........T

.........S.................................S.........T

......................................................

...............................................G......

...........................A..........................

Week 3 (7/26)

Week 3 (1/26)

Week 3 (1/26)

Week 3 (1/26)

Week 3 (1/26)

Week 3 (9/26)

Week 3 (1/26)

Week 3 (4/26)

Week 3 (1/26)

Week 4 (11/23)

Week 4 (10/23)

Week 4 (1/23)

Week 4 (1/23)

Additional file 2: Table S2c: Evolution of NS3 1317-1423 sequence in patient 3

1320

1325

1330

1335

1340

1345

1350

1355

1360

1365

1370

NS3

GEIPFYGKAIPIETIKGGRHLIFCHSKRKCDELAA*KLSGLGLNAV*AYYRGLDV

......................L...............................

..........................E...........................

.......................R..E...........................

.........................P............................

..........................RK..........................

..........................RK..........................

..........................RK.............I..I..........

..........................RK.........P................

..........................RK.............I..I.........

......................................................

......................................................

......................................................

Week 3 (7/26)

Week 3 (1/26)

Week 3 (1/26)

Week 3 (1/26)

Week 3 (1/26)

Week 3 (9/26)

Week 3 (1/26)

Week 3 (4/26)

Week 3 (1/26)

Week 4 (11/23)

Week 4 (10/23)

Week 4 (1/23)

Week 4 (1/23)

1375

1380

1385

1390

1395

1400

1405

1410

1415

1420

Additional file 2: Table S2c: Evolution of NS3 1317-1423 sequence in patient 3
